# *Toxoplasma gondii* Infection and Threatened Abortion in Women from Northern Peru

**DOI:** 10.1155/2022/1163655

**Published:** 2022-08-08

**Authors:** Mario J. Valladares-Garrido, Virgilio E. Failoc-Rojas, C. Ichiro-Peralta, David Astudillo-Rueda, Heber Silva-Díaz

**Affiliations:** ^1^South American Center for Education and Research in Public Health, Universidad Norbert Wiener, Lima, Peru; ^2^Hospital Regional Lambayeque, Lambayeque, Peru; ^3^Universidad San Ignacio de Loyola, Lima, Peru; ^4^Facultad de Medicina Hipólito Unanue, Universidad Nacional Federico Villarreal, Lima, Peru; ^5^Facultad de Medicina Humana, Universidad Cesar Vallejo, Piura, Peru; ^6^Factultad de Medicina Humana, Universidad de San Martín de Porres, Chiclayo, Peru

## Abstract

**Introduction:**

*Toxoplasma gondii* infection can cause important complications during pregnancy. Threatened abortion may be a late indicator for infection in settings with high prevalence of toxoplasmosis. We aimed to determine the association between *T. gondii* infection and threatened abortion in women from northern Peru.

**Methods:**

We conducted a secondary analysis of a cross-sectional study in pregnant women from a hospital and a rural community in Lambayeque, Peru. Exposure variable was serological diagnosis of toxoplasmosis, defined as the demonstration of either IgM or IgG antibodies against *T. gondii*. Outcome variable was threatened abortion, defined as the diagnosis of bloody vaginal discharge or bleeding during the first half of pregnancy. Prevalence ratios were estimated in simple and multiple regression analyses.

**Results:**

Of 218 pregnant women, 35.8% presented positive serology for *T. gondii* and 14.7% had threatened abortion in their current pregnancy. Pregnant women with positive *T. gondii* infection had 2.45-fold higher frequency of threatened abortion (PR: 2.45, 95% CI: 1.15-5.21). In addition, the frequency of threatened abortion decreased by 9% for each additional year of age (PR: 0.91, 95% CI: 0.86-0.97). A previous history of threatened abortion also showed a higher frequency of threatened abortion (PR: 5.22, 95% CI: 2.45-11.12).

**Conclusions:**

*T. gondii* infection is associated with threatened abortion. An early age of pregnancy and a previous history of abortion are also associated with this condition.

## 1. Introduction

*Toxoplasma gondii* is an intracellular protozoan capable of infecting a wide range of warm-blooded vertebrates [1]. Transmission of *T. gondii* occurs through the consumption of undercooked meat, unpasteurized milk from infected animals, contaminated water and vegetables, blood transfusions, organ transplants, and vertical transmission [2–4].

In pregnant women, the seroprevalence of *T. gondii* in Europe is reported to be between 9 and 48.7%, while in South America, it ranges between 38 and 77.5% [5]. In Peru, a seroprevalence of 39% has been reported [6]. There is a high risk of complications due to *T. gondii*, since it can cross the placental barrier and infect embryonic tissues [7]. More severe complications such as chorioretinitis, hydrocephalus, low birth weight, and central nervous system (CNS) abnormalities are associated with infections during the first and second trimester, while infection during the third trimester is associated with late congenital complications and developmental delay [8, 9]. Some studies have proposed *T. gondii* infection as a potential risk factor for miscarriage [10, 11].

Threatened abortion affects about 7-24% of the population [12, 13], and its occurrence may lead to the first trimester hemorrhage, vaginal infections, and shortening of cervical length, among others [14]. However, there is limited evidence supporting a possible association with *T. gondii* infection [15, 16]. The purpose of this study was to evaluate the association between *T. gondii* infection and the occurrence of threatened abortion in women from northern Peru.

## 2. Methods

### 2.1. Data Source

**Figure 1:**
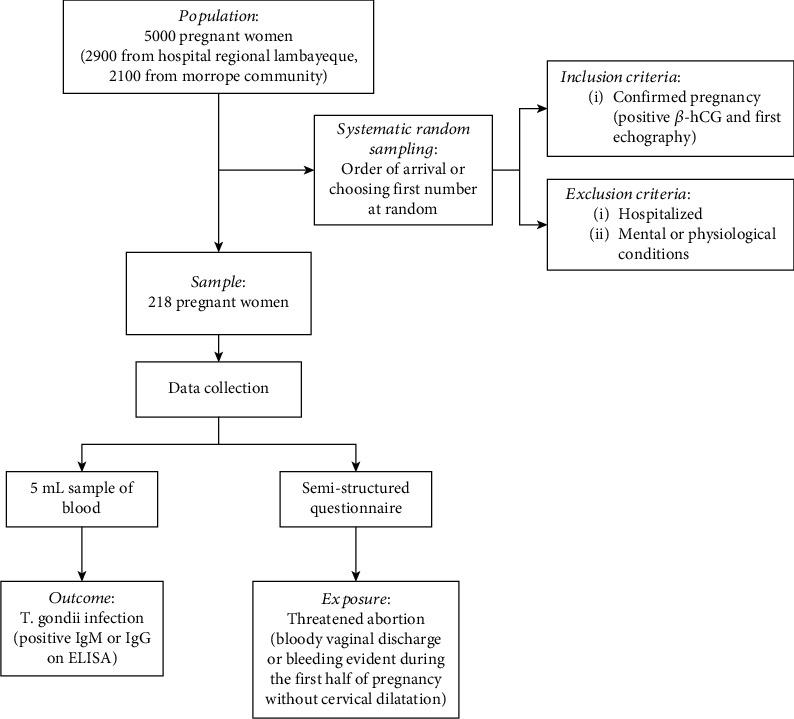
Flowchart of the data collection process.

We performed a secondary analysis based on data of a previous study [17]. This followed a cross-sectional design to identify the seroprevalence of toxoplasmosis in pregnant women in Lambayeque, a city in northern Peru. All the records of the primary study were used. A brief description of the data collection process is shown in Figure 1.

The population consisted of pregnant women who were treated at the Services of Obstetrics and Gynecology of Hospital Regional Lambayeque from July 2016 to June 2017 and pregnant women living in the district of Morrope, Lambayeque. A sample of 218 individuals was estimated from 2900 women from Hospital Regional Lambayeque and 2100 women from Morrope community, with an error of 0.06, an expected frequency of 30%, and a 95% confidence interval.

The data were collected using systematic random sampling based on the order of arrival to the hospital or nonhospital care services (Morrope community). Women with confirmed pregnancy (positive *β*-hCG and first echography) were included. A 5 mL sample of blood was collected using a vacuum system and a semistructured questionnaire was administered. The questionnaire was validated in the previous study and was divided into four sections: (1) sociodemographic information (eight questions), (2) clinical information (eight questions), (3) eating habits and environment (six questions), and (4) laboratory results (four questions). The last section was completed by a physician and biologist.

Positive serology was determined with the presence of serum anti-*T. gondii* antibodies in the sera stored at −70°C. The serological tests were standardized using enzyme-linked immunosorbent assay (ELISA), following the manufacturer's recommendations (Virion-Serion, Germany). Anti-*T. gondii* IgM and IgG antibodies and avidity of IgG were quantitatively evaluated. A positive interpretation occurred at values higher than 30 IU/mL of IgM and 350 IU/mL of IgG. Sensitivity and specificity of the kits were 97.8% and 95.7% for IgM and 98.2% and 99.4% for IgG, respectively.

Samples positive for anti-*T. gondii* IgG antibodies were qualified for the IgG avidity test. This was used to determine the time of seroconversion, suggesting a recent infection if avidity was lower than 45%. Seropositivity for toxoplasmosis was presumed when the tests were positive for one or more of the markers (IgM or IgG).

### 2.2. Variables

The exposure variable was *T. gondii* seropositivity, defined as a positive IgM or IgG antibody test using ELISA.

The outcome variable was a diagnosis of threatened abortion, defined as bloody vaginal discharge or bleeding evident during the first half of pregnancy without cervical dilatation. This was diagnosed by a gynecologist under clinical and ultrasound criteria.

Other covariates analyzed were age (years), area of residence (rural, urban), drinking water consumption (no, yes), consumption of raw vegetables (no, yes), history of threatened abortion (no, yes), family history of abortion (no, yes), number of pregnancies, and history of urinary tract infection during current pregnancy (no, yes).

### 2.3. Data Analysis

Absolute and relative frequencies were described for categorical variables. In the case of numerical variables, we reported the best measure of central tendency and dispersion, after evaluation of numerical and graphical normality.

In bivariate analysis, the association between *T. gondii* infection and threatened abortion was evaluated with the *χ*^2^ test for independence; the same was done with the other categorical covariables. For the variables age and number of pregnancies, Student's *t*-test and Mann–Whitney *U*-test were used, respectively.

Prevalence ratios (PRs) and 95% confidence intervals were calculated in simple and multiple regression analyses, adjusting for confounding variables. We used generalized linear models (GLM) with Poisson family of distribution, log link function, and robust variance. A significance level of 5% was considered. Data were analyzed in Stata version 15.0.

## 3. Results

Table 1 shows that most of the participants were from rural areas (56%) and the mean age was 26.4 years. A history of threatened abortion was reported by 22.9% of pregnant women. Only 37.6% reported consuming drinking water, while the majority reported consuming raw vegetables (89.9%). Less than half of participants had positive serology for *T. gondii* (35.8%). A total of 14.7% had threatened abortion in their current pregnancy.

*T. gondii*-seropositive pregnant women had a higher frequency of threatened abortion compared to *T. gondii*-seronegative pregnant women (20.0% vs. 11.8%; *p* = 0.131). Frequency of threatened abortion in pregnant women from urban areas was 13% higher than in those who lived in rural areas (23.1% vs. 10.1%; *p* = 0.017). Pregnant women with a previous history of threatened abortion were 25.1% more likely to have a threatened abortion in their current pregnancy compared to women without such a history (34.2% vs. 9.1%; *p* < 0.001). Pregnant women with a history of urinary tract infection in their current pregnancy had 18.8% higher frequency of threatened abortion compared to those without such a history (30.0% vs. 11.2%; *p* = 0.031).  Table 2.

In simple regression analysis, no statistical difference was observed between positive serology for *T. gondii* and threatened abortion. However, in multiple regression, it was found that pregnant women with positive serology for *T. gondii* were 1.45 times more likely to have a threatened abortion (PR = 2.45; 95% CI: 1.15-5.21), adjusted for confounding variables (area of residence, age, number of pregnancies, history of threatened abortion, family history of abortion, drinking water consumption, consumption of raw vegetables, and history of urinary tract infection during current pregnancy). The differences found in simple regression analysis were not significant in multiple regression analysis for area of residence, raw vegetable consumption, and urinary tract infection (*p* > 0.05). In addition, it was observed that for each additional year of age, the frequency of threatened abortion decreased by 9% (PR = 0.91; 95% CI: 0.86-0.97). Also, pregnant women with a previous history of threatened abortion were 4.22 times more likely to have threatened abortion (PR = 5.22; 95% CI: 2.45-11.12).  Table 3.

## 4. Discussion

We found that 20% of pregnant women with positive serology for *T. gondii* had threatened abortion, and both variables were positively associated when controlling for confounders. Additionally, age and previous history of abortion were positively associated with threatened abortion during current pregnancy.

We found that the prevalence of threatened abortion was 14.7%. This result is similar to a study in Korean women, in which the prevalence of threatened abortion was 14.8% [18]. This differs from that reported in Nigeria, in which the prevalence was 5.7% [19], and that reported in China, in which the prevalence was higher (23.4%) [20]. The differences found among these studies could be attributed to ethnic variation, as reported by Jones and Kavanaugh, in which a lower frequency of abortion in African-American and Hispanic pregnant women was found compared to Asian women [21].

The frequency of threatened abortion was 1.45 times higher in seropositive pregnant women compared to those with negative test results. It is known that in women with acute infection, the risk of abortion is higher in IgM-positive women than in those with negative IgM [22, 23]. However, other studies found no association between spontaneous abortion and the presence of IgG antibodies against *T. gondii* [22–24]. These studies evaluated spontaneous abortions as an outcome. However, we did not find any studies that specifically evaluated threatened abortion.

We found that the frequency of threatened abortion decreased by 9% for each additional year of age. This means that the older the maternal age, the lower the frequency of this adverse outcome during pregnancy. It has been similarly reported in other studies [25, 26] but with other obstetric adverse events, such as spontaneous abortion, fetal death, infant death, preterm delivery, cesarean section, and low birth weight, among others. However, research has not found differences in threatened abortion and other complications during pregnancy or childbirth [22, 25–27]. Conversely, our findings differ from those reported by Magnus et al. [28], which found a higher risk of abortion in women over 30 years of age (reaching a peak of 57%). It is observed that the risk of abortion during pregnancy occurs in two specific stages of maternal age: in adolescents under 19 years of age and in women over 30 years of age (J-curve phenomenon) [28–30]. This can be explained by the physiological immaturity of the uterus in the first stage, and, conversely, a decline in uterine and hormonal function in the second stage [31]. It is also known that advanced maternal age is associated with a greater number of cytogenetic alterations [32].

A previous history of abortion was associated with a higher frequency of threatened abortion during current pregnancy. This result is consistent with what has been observed in previous studies [33, 34]. However, McPherson found that women with a history of multiple second trimester pregnancy losses were associated with an increased risk of late miscarriage and fetal death, but not with recurrent first trimester miscarriage [35, 36], a phenomenon that could be explained by genetic and hemostatic factors. The influence of age on the risk of recurrent abortion can also be considered, given that the attempt of new pregnancies will necessarily occur at older ages.

### 4.1. Limitations

The main limitation of this study is the lack of temporality related to its cross-sectional design, meaning that it is not possible to attribute a causal effect of *T. gondii* infection on the occurrence of threatened abortion. Also, there is a potential response bias, given that sociodemographic variables were collected by self-report. Other important variables were not included in the study (e.g., consumption of undercooked meat and recurrent abortion), which may lead to inaccurate estimates of association. Finally, the study is limited to a specific region of Peru, so it is not possible to infer the results in all Peruvian pregnant women. Nevertheless, we have evidenced an underlying problem in Peru, providing useful information for future research and public health policy in the region.

## 5. Conclusions

Pregnant women infected with *T. gondii* have higher rates of threatened abortion. We encourage further research on this topic to support the development of effective interventions for *T. gondii* control and early diagnosis of infection in pregnant women (and those planning pregnancy).

## Figures and Tables

**Table 1:** Characteristics of pregnant women attended in a hospital in Lambayeque, Peru.

Characteristics	*n* (%)
Area of residence	
Rural	122 (56.0)
Urban	96 (44.0)
Age (years)^∗^	26.4 ± 6.86
Number of pregnancies^∗∗^^†^	2 (1-10)
History of threatened abortion	
No	168 (77.1)
Yes	50 (22.9)
Family history of abortion^†^	
No	153 (86.0)
Yes	25 (14.0)
Drinking water consumption	
No	136 (62.4)
Yes	82 (37.6)
Consumption of raw vegetables	
No	22 (10.1)
Yes	196 (89.9)
History of urinary tract infection^†^	
No	162 (88.5)
Yes	21 (11.5)
*T. gondii* seropositivity^††^	
No	140 (64.2)
Yes	78 (35.8)
Threatened abortion in current pregnancy^†^	
No	157 (85.3)
Yes	27 (14.7)

^∗^Mean ± standard deviation. ^∗∗^Median (minimum value to maximum value). ^†^Some values do not add up to 218 due to missing data. ^††^*T. gondii* seropositivity includes either an IgM- or IgG-positive ELISA test.

**Table 2:** *T. gondii* seropositivity and other factors associated with threatened abortion in pregnant women, bivariate analysis.

Variables	Threatened abortion		*p* value
	No (*n* = 157)	Yes (*n* = 27)	
	*n* (%)	*n* (%)	
Area of residence			0.017^∗^
Rural	107 (89.9)	12 (10.1)	
Urban	50 (76.9)	15 (23.1)	
Age (years)^¶‡^	26.3 ± 7.22	25.4 ± 5.99	0.565
Number of pregnancies^†§‡‡^	2 (1-10)	2 (1-7)	0.827
History of threatened abortion			<0.001^∗^
No	130 (90.9)	13 (9.1)	
Yes	27 (65.9)	14 (34.2)	
Family history of abortion^†^			0.144^∗∗^
No	131 (86.2)	21 (13.8)	
Yes	24 (96.0)	1 (4.0)	
Drinking water consumption			0.628^∗^
No	97 (84.4)	18 (15.7)	
Yes	60 (87.0)	9 (13.0)	
Consumption of raw vegetables			0.064^∗∗^
No	11 (68.8)	5 (31.3)	
Yes	146 (86.9)	22 (13.1)	
History of urinary tract infection during pregnancy^†^			0.031^∗∗^
No	143 (88.8)	18 (11.2)	
Yes	14 (70.0)	6 (30.0)	
*T. gondii* seropositivity			0.131^∗^
No	105 (88.2)	14 (11.8)	
Yes	52 (80.0)	13 (20.0)	

^‡^Mean and standard deviation. ^‡‡^Median (minimum value to maximum value). ^†^Some values do not add up to 218 due to missing data. ^∗^*p* values calculated with *χ*^2^ test for independence. ^∗∗^*p* values calculated with Fisher's exact test. ^¶^*p* value calculated with Student's *t*-test for constant variances. ^§^*p* value calculated with Mann–Whitney *U* test.

**Table 3:** Association between *T. gondii* seropositivity and threatened abortion in pregnant women, simple and multiple regression analysis.

Characteristics	Simple regression			Multiple regression∗		
	PR	95% CI	*p* ^∗∗^	PR	95% CI	*p* ^∗∗^
Area of residence						
Rural	Ref.			Ref.		
Urban	2.29	1.14 - 4.60	0.020	0.99	0.40 - 2.48	0.985
Age (years)	0.99	0.94 - 1.03	0.515	0.91	0.86 - 0.97	0.006
Number of pregnancies	1.01	0.83 - 1.23	0.928			
History of threatened abortion						
No	Ref.			Ref.		
Yes	3.76	1.92 - 7.36	<0.001	5.22	2.45 - 11.12	<0.001
Family history of abortion						
No	Ref.			Ref.		
Yes	0.29	0.04 - 2.07	0.217	0.33	0.04 - 2.59	0.290
Drinking water consumption						
No	Ref.			Ref.		
Yes	0.83	0.40 - 1.75	0.631	0.81	0.33 - 2.00	0.643
Consumption of raw vegetables						
No	Ref.			Ref.		
Yes	0.42	0.18 - 0.96	0.039	0.47	0.17 - 1.29	0.143
History of urinary tract infection during current pregnancy						
No	Ref.			Ref.		
Yes	2.68	1.20 - 5.98	0.016	1.29	0.50 - 3.30	0.595
*T. gondii* seropositivity						
No	Ref.			Ref.		
Yes	1.70	0.85 - 3.40	0.134	2.45	1.15 - 5.21	0.020

^∗^Adjusted for area of residence, age, number of pregnancies, history of threatened abortion, family history of abortion, drinking water consumption, consumption of raw vegetables, and history of urinary tract infection during pregnancy. ^∗∗^*p* values obtained with generalized linear models with Poisson family of distribution, log-link function, and robust variance.

## Data Availability

The data used to support the findings of this study are available from the corresponding author upon request.
